# Introducing advanced surgical tasks simulation for surgical training

**DOI:** 10.1016/j.amsu.2022.103568

**Published:** 2022-04-04

**Authors:** Faiz Tuma, Lisa M. Knowlton, Aussama K. Nassar

**Affiliations:** a Central Michigan University College of Medicine, United States; b Western Michigan University M.D. Home Stryker School of Medicine, United States; c Stanford University Medical Center, United States

**Keywords:** Advanced surgical skills, Simulation, Surgical evaluation, Surgical training

## Abstract

Acquiring surgical skills is one of the major objectives of surgical training. Trainees face increasing challenges to meet the continuously evolving surgical techniques and approaches during the limited time course of their surgical training. The limited availability of training tools for teaching advanced surgical skills is an additional barrier. Educators have increasingly used simulation tools for surgical skills training around the globe. However, current simulation training modules and curricula focus mainly on basic surgical skills. Hence, the development of advanced virtual simulation modules offers a precious laparoscopic training opportunity. This article provides an educational technology-based review and proposal (with selected examples) of simulation training modules on advanced surgical skills that can be used for advanced surgical training approaches.

## 1 Introduction

Advanced surgical skills teaching poses an increasing challenge for trainees, instructors, and their training programs as it requires intensive psychomotor deliberate practice [1]. Given operative resource constraints, increasing patient volume, and focus on patient safety; this deliberate practice is ideally performed in a simulated environment [1]. However, current technical skills training outside the operating room is focused on relatively basic surgical skills acquisition, with a few exceptions. Simulation training on advanced technical skills such as dissecting between planes or isolating delicate structures has been limited, making learning those skills on patients in the operating room potentially unsafe and inefficient. This limitation imposes substantial demand for curricula that address a comprehensive surgical skills training spectrum to meet their specific educational needs [2]. However, various challenges exist in this direction, such as the mentor's availability, resources and training opportunities [3,4], the pace of skill acquisition of trainees, and the training environment. Hence, surgical trainees often undergo a long and unstructured learning curve to acquire these advanced skills leading to unpredictable training outcomes. It is, therefore, necessary to introduce more practical, structured training modules that provide graduated, predictable, and assessable training supported by the concept of the incremental acquisition of skills [2]. The objective of this article is to introduce training on advanced laparoscopic skills via simulation and to describe a few examples and methodologies that facilitate implementation.

### 1.1 Surgical simulation educational technology

Surgical education technology provides a reliable alternative to training opportunities that put patients at risk [1]. Several studies have shown that trainees that undergo surgical simulation training demonstrate skill improvement, including decreased time of operations, reduced complications, and the likelihood of progression of the procedure [2,5]. With technology, educators can create various digital and hands-on learning experiences tailored to their educational needs [6]. Virtual simulation has been used to train surgical residents and students on many simple and minimally invasive surgical procedures. However, currently available standardized simulation modules are designed and structured to teach simple tasks such as clipping a vessel, moving an object, cutting a structure, and other low-level procedures. A few advanced training modules such as laparoscopic suturing exist but limitedly. Therefore, there is a strong need to develop and implement advanced skills training simulation. We propose a few commonly performed advanced laparoscopic techniques that trainees should acquire before applying to patients. These modules are designed and structured according to the educational principles of psychomotor and cognitive skill [2,7,8] training and evaluation. Various modules have been developed and discussed in the literature. This article presents and discusses several surgical tasks that are routinely performed and require advanced skill sets.

### 1.2 Proposed laparoscopic advanced virtual simulation modules

#### 1.2.1 Dissection between planes

Dissecting between planes model is used to train and evaluate various operative skills such as dissecting around the gastro-esophageal junction, reducing the hernial sac in a hernia repair, and mobilizing the colon in preparation for colon surgeries.

##### 1.2.1.1 Task steps and structure

1. Identify the two planes in different models and evaluate the degree of difficulty of dissection.
2. Identify the safe plane separating the two structures.
3. Dissect bluntly between the structures where it is easier to do so.
4. Wider dissection and maintaining a broad exposure before extending the dissection deeper.
5. Dissect parallel to the tissue planes initially, not perpendicular.
6. Handle tissue gently, especially with delicate side/plane, e.g., bowel.
7. Avoid blunt dissection at dense bands or adhesions. Instead, dissect bluntly on both sides of these dense adhesions then dissect them sharply with scissors or electro-cautery.
8. Avoid dissecting or cutting thick tissue bundles without exploring them. These bundles may contain important structures – such as blood vessels – that need special handling. Instead, dissect these bundles using 360-degree visualization where possible, then carefully explore the bundle before deciding where to make incisions.

##### 1.2.1.2 Evaluation of performance

1) The economy of movements (number of movements); 2) the precision of movements (straight, smooth movements); 3) the direction of incision (parallel vs. perpendicular to tissue planes). 4) the ratio of depth and width of incision. 5) the presence of excessive tension or stretching on tissue; 6) the presence of injury to vital structures; and 7) the time needed to complete the task.

#### 1.2.2 Mobilizing organs/structures

Mobilizing the colon, the kidneys, and the duodenum are examples of mobilization, an essential surgical skill. A training and evaluation model can be structured to acquire this competency outside the OR.

##### 1.2.2.1 Task steps and structure

1. Identify anatomy landmarks, boundaries, attachments, and pedicles of the various organs.
2. Review and determine the direction, sides and sequence, and the endpoints of mobilization.
3. Dissect using both sharp (scissors, knife, cautery) and blunt (suction tip, grasper tips spread, sponge, etc.) dissection models.
4. Identify the avascular optimal plane in the correct direction toward the endpoint.
5. Avoid injuring the organ tissue and supplying neuro-vascular pedicles.
6. Achieve various degrees of hemostasis.
7. Finalize the mobilization safely to the endpoint.

##### 1.2.2.2 Evaluation of performance

1) Appropriate starting steps and sites of mobilization; 2) the economy of movements (number of moves); 3) the precision of movements; 4) the presence of excessive tension or stretching on tissue; 5) the presence of injury to vital structures; and 6) the time needed to complete the task.

#### 1.2.3 Lysis of adhesions

Application of the lysis of adhesions procedure is required to release small bowel adhesions within the abdominal wall or between the loops and prepare for incisional hernia repair with multiple adhesions. Training on this core surgical skill can be achieved with a model that addresses all aspects of the skill and its levels.

##### 1.2.3.1 Tasks steps and structure

1. Identify the anatomy and extent of the adhesions, finding the avascular adhesion points and planes.
2. Ensure cautious exploration, exclusion of vital structures, and gentle tissue handling.
3. Lyse adhesions to release the affected (possibly compromised) segment and the attached surrounding structures.
4. Ensure cautious use of energy devices with adequate blunt and sharp dissection techniques.
5. Avoid performing excessive lysis of adhesions of the unaltered segments.
6. Recognize and adequately repair serosal tears.
7. Finalize release of adhesions to the predetermined target.

##### 1.2.3.2 Evaluation of performance

1) Appropriate visualization of the adhesions and effective recognition of the affected areas; 2) appropriate application of traction and counter traction before dissecting the adhesions; 3) adequate hemostasis; 4) the economy of motion; 5) the precision of movements; 6) appropriate dissection of the altered segment requiring release; 7) avoidance of injury of the bowel or important structures; and 8) proper identification and repair of serosal tears

#### 1.2.4 Running the small bowel

Running the small bowel could be an adjunct skill for several types of surgeries where the evaluation of the entire bowel for possible ischemia or other abnormalities is needed. Therefore, it is imperative to conduct an adequate assessment of the small bowel while ensuring safe tissue handling to avoid overlooking key findings.

##### 1.2.4.1 Tasks steps and structure

1. Identify the terminal ileum close to the ileocecal valve.
2. Using two non-crushing graspers (in the laparoscopic approach), hold the small bowel segments vertically, about 12–15 cm apart.
3. Identify bowel and mesenteric surfaces on both sides of the small bowel segment.
4. Advance bowel by releasing the distal end and holding it proximal to the proximal hand with the same distance of 12–15 cm in between.
5. Evaluate the new proximal segment in the same way.
6. Complete the entire length of the small bowel up to the Treitz ligament.

##### 1.2.4.2 Evaluation of performance

1) Effective handling of the bowel using adequate instruments and traction; 2) appropriate identification of the affected segment; 3) identification of the entire segment completed before moving forward; 4) the economy of movements; 5) the precision of movements; 6) always maintaining laparoscopic instruments within view; 7) avoidance of injury of the bowel or other vital structures.

#### 1.2.5 Control of small vessel bleeding

Control of laparoscopic bleeding is an important adjunct skill to any procedure and can prove challenging. This skill is particularly useful in larger caliber vessel bleeds, such as bleeding from the cystic or gonadal vessel.

##### 1.2.5.1 Task steps and structure

1. Visually identify the bleeding without moving retraction.
2. Suction the area with a large-bore suction device.
3. Apply an atraumatic grasper to the identified bleeding point.
4. Place a mechanical clip or tie on both sides of the area being grasped.
5. Irrigate and evaluate.

##### 1.2.5.2 Evaluation of performance

1) The time taken to identify the bleeding vessel; 2) Using suction to identify the bleeding site; 3) the time taken to grasp the bleeding site; 4) positioning of the clip used for hemostasis; 5) achieving control of bleeding; 6) the economy of motion; and 7) the precision of movements.

Trainees should be evaluated before and after specific periods of training. The objective of these evaluations is to significantly decrease the time required for the completion of a particular task and obtain an objective assessment by a faculty member. In addition, these objective evaluations would aim to see significant improvement in subsequent cases performed by the resident.

## 2 Discussion

The surgical practice continues to evolve and integrate more challenging procedures that require the development of technical skills training [9]. Since the development of the fundamentals of laparoscopic skills (FLS), there has been a growing need for a simulated advanced laparoscopic surgical skills curriculum. Studies have shown a significant difference in performance after FLS and advanced laparoscopic skills (ALS) training on trainees, and faculty [10]. Acquiring surgical skills can be enhanced with advanced virtual simulation modalities. Training on advanced surgical skills is often unpredictable in the operating room (OR). Therefore, developing simulation modules for these skills will narrow the gap between simulation training and real-life surgical practice. Novel training approaches are necessary to establish surgical competency [11].

Psychomotor skills training poses substantial challenges to skill acquisition at the advanced level [2]. Efficient training tools are needed to meet these challenges. Simulation of advanced skills facilitates skill acquisition and provides further insight into how trainees acquire these skills. The trainees' progress through these simulation modules provides insight into the learning process and skill acquisition. Data on the improvement of both cognitive surgical skills and psychomotor skills can be evaluated and analyzed to better understand the nature of advanced surgical skill acquisition. This knowledge can then be applied to training on patients to improve training efficacy.

The simulation of advanced surgical skills allows developing psychomotor and technical skills required for competent surgical performance in a safer environment than traditional training [9]. In addition to providing valuable training tools [4] on advanced surgical skills and opportunities to learn about how trainees acquire these skills, these advanced training modules offer a valuable way to evaluate trainees' performance. Advanced skill sets require a specialized evaluation technique [12]. The required techniques involve similar stages and components of the advanced training modules. Hence, utilizing these modules to evaluate advanced skills objectively presents a valuable evaluation tool since the currently used tools are mostly subjective [13].

## 3 Limitations

Like most novel projects, implementation will be challenging for various reasons. The required infrastructure and budgets to make these modules available for training are beyond the scope of many institutions. Hence, the surgical training industry may be the best option. However, before investing in such a project, the industry will require validation of this training approach. Therefore, the next step would be studying the practicality and validity of the approach. Face validity can be assessed by examining educators' opinions and perspectives. In comparison, content validity can be assessed by experiments on a model constructed for that purpose.

## 4 Conclusions

Surgical skills training can be enhanced with the addition of advanced skills training virtual simulation modules. Current educational technologies provide the tools and means of application and dissemination. Additional steps to apply and validate these modules are required to validate and implement these models.

## Sources of funding

The authors report no funding

## Ethical approval

This is a review study on education that involves no patient identifiers. Therefore, no ethical approval or consent were needed.

## Consent

This is a review study of education that involves no human beings. Therefore, no ethical approval or consent were needed.

## Author contribution

**FT** Conceptualization, writing original draft, editing & reviewing, visualization, project administration. **LK** Writing original draft, editing & reviewing, visualization, project administration. **AN** Writing original draft, editing & reviewing, visualization, project administration.

## Registration of research studies

This is a review study that involves no experiment or data to register.

## Guarantor

Faiz Tuma MD, MME, MDE, EdS.

## Provenance and peer review

Not commissioned, Editor reviewed.

## Declaration of competing interest

The authors report no conflicts of interest.
